# Chemical and Structural Characterization of Maize Stover Fractions in Aspect of Its Possible Applications

**DOI:** 10.3390/ma14061527

**Published:** 2021-03-20

**Authors:** Magdalena Woźniak, Izabela Ratajczak, Dawid Wojcieszak, Agnieszka Waśkiewicz, Kinga Szentner, Jacek Przybył, Sławomir Borysiak, Piotr Goliński

**Affiliations:** 1 Department of Chemistry, Faculty of Forestry and Wood Technology, Poznań University of Life Sciences, 60625 Poznań, Poland; magdalena.wozniak@up.poznan.pl (M.W.); agnieszka.waskiewicz@up.poznan.pl (A.W.); kinga.szentner@up.poznan.pl (K.S.); piotr.golinski@up.poznan.pl (P.G.); 2 Department of Biosystems Engineering, Faculty of Environmental Engineering and Mechanical Engineering, Poznań University of Life Sciences, 60627 Poznań, Poland; jacek.przybyl@up.poznan.pl; 3 Institute of Chemical Technology and Engineering, Poznan University of Technology, 60965 Poznań, Poland; slawomir.borysiak@put.poznan.pl

**Keywords:** maize stover fractions, chemical composition, cellulose, lignin, FTIR, supermolecular structure, crystallinity

## Abstract

In the last decade, an increasingly common method of maize stover management is to use it for energy generation, including anaerobic digestion for biogas production. Therefore, the aim of this study was to provide a chemical and structural characterization of maize stover fractions and, based on these parameters, to evaluate the potential application of these fractions, including for biogas production. In the study, maize stover fractions, including cobs, husks, leaves and stalks, were used. The biomass samples were characterized by infrared spectroscopy (FTIR), X-ray diffraction and analysis of elemental composition. Among all maize stover fractions, stalks showed the highest C:N ratio, degree of crystallinity and cellulose and lignin contents. The high crystallinity index of stalks (38%) is associated with their high cellulose content (44.87%). FTIR analysis showed that the spectrum of maize stalks is characterized by the highest intensity of bands at 1512 cm^−1^ and 1384 cm^−1^, which are the characteristic bands of lignin and cellulose. Obtained results indicate that the maize stover fraction has an influence on the chemical and structural parameters. Moreover, presented results indicate that stalks are characterized by the most favorable chemical parameters for biogas production.

## 1. Introduction

The constantly increasing and improving world production of maize is followed by an increase in crop residue volume. Combine harvesting of maize allows the grain to be separated from the other fractions of the plant-residues, called stover, consisting of cobs, husks, stalks, and leaves [1]. Obtained biomass is heterogeneous and constitutes about 50% of the dry weight of whole plants. Nevertheless, the approximate proportion of individual stover components was determined, and for 1 kg of dry maize grains, these components include (in kg) about 0.50 of stalks, 0.22 of leaves, 0.15 of cobs, and 0.14 of husks [2,3].

Crop residues have various applications, often with the use of individual fractions; for example, leaves can be used in paper production as a good source of fiber and sugars, bio-fertilizers can be obtained from stems, leaves and husks, while building materials from maize cobs [4,5,6]. Maize cobs and stalks have been used in the production of particleboards, while husks have been used in the preparation of low-density polyethylene composites [7,8,9]. In addition, according to literature data, cellulose or nanocellulose can be produced from maize crop residues [10,11]. Moreover, maize stover fractions can also be applied as substrates for the cultivation of oyster mushrooms or a feed source of ruminants [12,13]. In turn, maize stover left in the field protects the soil and improves its quality.

In the last decade, an increasingly common method of maize stover management is to use it for energy production, including the production of bioethanol, biochar or biogas [14,15,16]. Maize crop residues have been shown to have great potential as alternative plant raw materials for the anaerobic digestion (AD) for biogas production [17,18,19].

The AD technology allows for the production of biogas in the process of organic matter degradation. A particular advantage of this form of renewable energy production is the ability to recover crop residues used in the fermentation process and return them back to the soil, thus preventing its depletion in an organic matter [14].

Effective utilization of maize stover requires a thorough knowledge of the structure of tissues, fibers and the chemical composition of individual fractions. The main component of the plant cell wall, lignocellulosic biomass, consists of three fundamental components: cellulose, hemicellulose and lignin [20], the last being the most complex of the three-dimensional amorphous biopolymers, encapsulating the other two components through hydrogen and covalent bonds [21,22,23]. Additionally, due to the ordered crystalline areas found in cellulose, it is highly stable and thus scarcely degradable [24]. The complex lignocellulosic material is considered by most authors as a whole, without studies on the composition of its particular fractions. However, in order to use the most favorable fractions for biogas production, prototypes of harvesting machines are designed, prepared to separate maize stover at this stage [14]. Such an approach would facilitate the fermentation process and increase its efficiency. Additionally, in order to further enhance the efficacy of the process, the pretreatment of lignocellulosic material is also applied.

The objective of this study was to provide chemical and structural characteristics of maize stover fractions, namely cobs, husks, leaves and stalks, and based on these parameters, to evaluate the potential application of these fractions. In this article, we focused mainly on the use of the maize stover fraction as a feedstock for the production of methane. Our goal was to find a correlation between the chemical and structural parameters (including carbon, hydrogen, nitrogen, sulfur and oxygen (CHNSO) concentrations, cellulose and lignin contents and the degree of crystallinity) of the maize stover fractions and their use in biogas production. The relationship between the chemical parameters, as well as crystallinity and the maize stover fractions, was also evaluated. This paper is the first report presenting the influence of chemical parameters and the supermolecular structure of maize stover fractions on their use in biogas production. The results presented in this paper are of considerable scientific and applicatory value. Literature sources presented data concerning methane efficiencies and cellulose content of maize stover fractions [3,14]. However, the lignocellulosic complex less susceptible to degradation has not been investigated in detail. At present, there are technical possibilities to harvest maize stover fractions separately [3]. Therefore, the results presented in this paper can be interesting to biogas plant operators looking for economically and technologically optimal substrates.

## 2. Materials and Methods

### 2.1. Maize Stover Fractions

The experimental materials were fractions (cobs, husks, leaves and stalks) of maize stover of cv. Podium (FAO 200, Warsaw, Poland). The final density before the harvest was 75 thousand plants per hectare. Both maize grain and the maize stover fraction were harvested in October 2017. The plants for analysis were collected manually by cutting 10 cm above the ground. Then they were manually divided into four fractions.

The next step in sample preparation consisted of their drying at 60 °C. The dry samples were ground three times, which included first pre-grinding followed by fine double-grinding.

### 2.2. Chemical Analysis

#### 2.2.1. CHNSO Concentration Analysis

The concentrations of basic elements (nitrogen, hydrogen, carbon, oxygen and sulfur) in samples were determined using a Flash 2000 elemental analyzer (Thermo Fisher Scientific, Waltham, MA, USA) according to EN ISO 16,948:2015 [25]. The instrument for CHNS determination was calibrated with (2,5-bis-(tert-butyl-benzoxazole-2-yl)thiophene) (Thermo Fisher Scientific, Waltham, MA, USA) and birch leaf (Elemental Microanalysis Ltd., Okehampton, UK). Benzoic acid (Thermo Fisher Scientific, Waltham, MA, USA) was used for calibration in oxygen determination. For each element, the six-point calibration curves were plotted using the K factor as the calibration method.

#### 2.2.2. Cellulose and Lignin Content

The material of maize stover fractions used for the analysis of cellulose and lignin content was previously extracted in ethanol according to the TAPPI method [26]. The cellulose content was determined according to the Seifert method using the mixture of acetylacetone, 1,4-dioxane and concentrated hydrochloric acid [27]. The prepared mixture with 1 g lignocellulosic material was heated for 30 min in a water bath at 100 °C. Then the samples were filtered and washed successively with methanol, dioxane, hot water and methanol. The lignin content was determined according to the TAPPI method using concentrated 72% sulfuric acid to hydrolyze and dissolve polysaccharides [28]. The samples of cellulose and lignin were dried at 102 ± 3 °C. Three replications were performed for all determinations. The obtained components of the maize stover fractions were used in infrared spectroscopy FTIR (cellulose and lignin) and X-ray diffraction XRD (cellulose) analyses.

#### 2.2.3. X-ray Diffraction Analysis

The supermolecular structure of biomass samples and cellulose isolated from this material was analyzed by means of X-ray diffraction (TUR-M62 diffractometer, Carl Zeiss, Jena, Germany). Both types of materials (biomass and isolated cellulose) for X-ray investigations were prepared in the form of compressed pellets following the principle of powder diffraction. The operating conditions of the diffractometer were: the wavelength of the copper K_α_ radiation source (1.5418 Å), current (30 mA), voltage (40 kV). The diffraction pattern was recorded in the range of 5 and 30 (2θ). The counting step of 0.04°/3 s was applied. The process of diffraction maxima deconvolution was carried out following the method described by Hindeleh and Johnson [29] and programmed by Rabiej [30]. After the deconvolution process, the degree of crystallinity (Xc) was calculated by comparing areas under the crystalline peaks and the amorphous curve.

#### 2.2.4. Fourier-Transform Infrared Spectroscopy (FTIR)

The FTIR spectra of biomass and its main components, i.e., cellulose and lignin, were obtained using a Nicolet iS5 spectrophotometer with Fourier-transform (Thermo Fisher Scientific, Waltham, MA, USA). The tested samples (1 mg) were mixed with 200 mg potassium bromide (Sigma-Aldrich, Darmstadt, Germany) and pressed to form tablets for the analyses. The spectra (32 scans) of the tested samples were recorded in a range of 4000–5000 cm^−1^ at a resolution of 4 cm^−1^.

### 2.3. Statistical Analysis

Statistical analyses included factorial ANOVA, followed by Tukey’s honest significant difference (HSD) test at α = 0.05. The Kendall rank correlation coefficient calculations were also performed between the factors. The statistical analysis was conducted using the STATISTICA 13.1 software.

## 3. Results and Discussion

**Figure 1:**
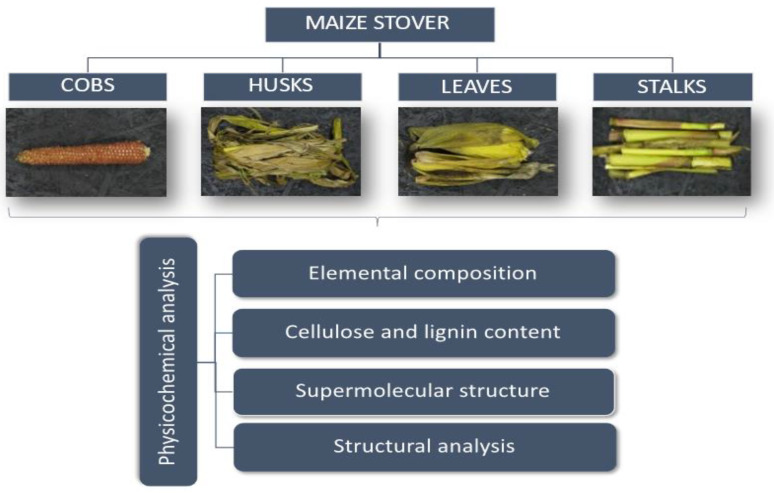
The scheme for characterization of maize stover fractions.

The composition of individual maize stover fractions (cobs, husks, leaves and stalks) was analyzed by the chemical method. The characterization scheme of the maize stover fractions is presented in Figure 1.

### 3.1. Chemical Composition

A comprehensive analysis of all the maize stover fractions and the C:N ratio is given in Table 1. The statistical analysis revealed that the percentage contents of carbon, nitrogen and oxygen depend on the maize fraction, while the content of hydrogen in all the fractions was statistically non-significant. Maize cobs contained the highest amount of carbon among all the fractions of maize, which was confirmed by ANOVA analysis. Sulfur contents in all the maize stover fractions were below the detection limit of the elemental analyzer (≤0.01%), which in turn results in small amounts of hydrogen sulfide gas produced or sulfur oxides emitted during the gasification process [31]. The nitrogen content in the maize stover fractions was 0.30–0.96%, with differences in particular fractions confirmed by ANOVA analysis. The oxygen content was the highest in maize cobs (45.03%), while it was lowest in maize stalks (41.35%), followed by leaves (41.91%).

The literature typically presents the elemental composition of whole maize plants or maize cobs only, whereas the results for the other individual fractions are rarely given. Carbon content in stalks of tested maize (43.73%) was lower than in the stalk shell (47.87%) and stalk pith (44.69%) of maize from those reported from Story Country, IA, USA [32]. Nitrogen contents in stalks (0.30%) and leaves (0.96%) of studied maize were lower when compared to those in the stalk (0.95–1.04%) and leaves (1.21%) of maize from Henan, China [33]. The percentage content of carbon in cobs of tested maize (44.8%) was similar to that of maize from Malaysia (43.8%) and lower than in maize cobs harvested in Turkey (49.0%), Serbia (47.6%), Hawaii (47.0%) and China (48.1%) [13,34,35,36,37], while N content in tested maize cobs (0.5%) was comparable to the level in maize cobs from Turkey (0.5%), Serbia (0.6%), and Hawaii (0.5%) and lower than in maize cobs from Malaysia (0.8%) or China (1.9%) [13,34,35,36,37]. The H content in maize cobs from Poland (5.9%) was higher than from Turkey (5.3–5.6%) and lower than from Malaysia (6.5%), Serbia (6.3%), Hawaii (6.4%) and China (6.5%) [13,34,35,36,37]. On the other hand, the oxygen content in cobs of the studied maize (45.0%) was higher than in Turkish (43.8–44.7%), Serbian (43.9%), Hawaiian (43.9%) and Chinese (43.5%) maize cobs and lower than in Malaysian cob samples (48.2%) of maize [13,34,35,36,37]. Maize stover fractions differed in their elemental composition, which in turn is related to climatic conditions, the variety of maize, harvest time and soil type [38,39,40,41]. Therefore, it seems important to study the relationship between the composition and chemical structure of individual maize fractions, as it is important when designing technological processes for maize waste processing, including balance calculations related to the biogas fermentation process.

The type of carbon source has an effect on anaerobic digestion by supporting different groups of microbes [42]. Next to carbon, also nitrogen content is another important factor in the methane production process. Nitrogen is the basic component of amino acids used in the synthesis of proteins; therefore, it is essential for the growth of microorganisms [42,43]. The carbon-to-nitrogen ratio in biomass significantly influences methane fermentation [44]. An excessively high C:N ratio causes acidogenic bacteria to consume nitrogen quickly compared to methanogenic bacteria. In turn, when the C:N ratio is too low, the microorganisms quickly consume nitrogen for growth. This causes nitrogen to accumulate in the form of ammonium ions, which increases the pH, in turn adversely affecting the production of biogas [42,43]. The highest C:N ratio was observed for stalks (147), the lowest for leaves (45), while for husks and cobs, it was 83 and 84, respectively, with the above fractions forming homogeneous groups in terms of their C:N ratios. The C:N ratio in tested cobs (84) was lower than in cobs harvested in Hawaii (94) and Turkey (98) and higher than in maize from Malaysia (57), Serbia (79) and China (25) [13,34,35,37].

**Figure 2:**
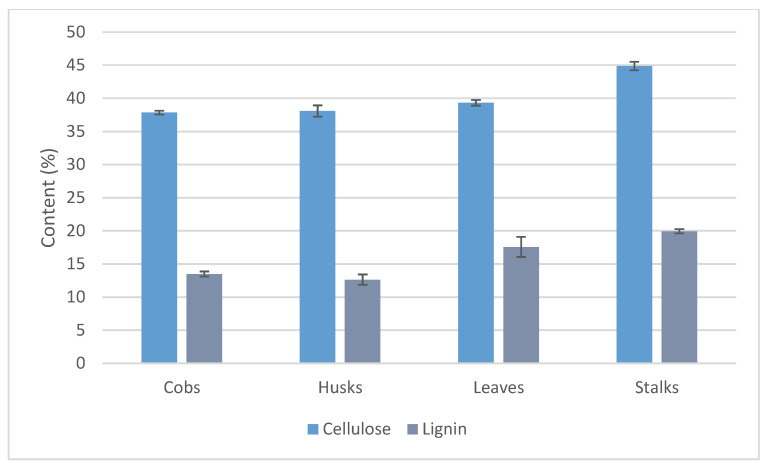
Cellulose and lignin contents in maize stover fractions according to [3].

The crucial factor in methane production is also related to the content of main biomass components, including cellulose and lignin. Cellulose and hemicellulose are biodegradable, whereas lignin, which is resistant to anaerobic bacteria, may reduce the methane yield [45,46,47]. The lignin and cellulose contents in the maize stover fractions (Figure 2) indicate that stalks contained higher amounts of cellulose than the other fractions, while lignin content in stalks was comparable to that in leaves and higher than in cobs and husks. Considering lignin content, two homogeneous groups were found for stalks and leaves versus cobs and husks; however, differences in the cellulose content in cobs, leaves and husks were statistically non-significant.

Stalks of tested maize contained higher amounts of lignin (19.9%) than stalks of Italian maize (8.0%), and the stalk fraction of maize from the USA (shell—13.5% and pith—6.1%) [14,32], while it was lower than in maize stalks from China (21.5%) [13]. The lignin content in husks (12.6%) and leaves (17.6%) of Polish maize was higher than that in husks (3.1%) and leaves (5.8%) of Italian maize [14]. The lignin content in cobs of tested maize (13.5%) was higher than in cobs of maize from Italy (4.8%), the USA (10.3%), Malaysia (11.3%) and comparable to yellow maize cobs from Nigeria (13.4%) [14,32,34,48]. In turn, cobs of maize from Turkey and China were characterized by higher amounts of lignin (15.5% and 19.6%, respectively) than cobs of Polish maize [13,35]. On the other hand, cellulose content in tested maize cobs (37.9%) was comparable to that of maize from Italy (36.4%) and lower than in maize from Turkey (52.0%), the USA (45.2%) and Malaysia (45.9%) [14,32,34,35]. The content of cellulose in maize cobs (37.9%) was higher than in cobs of white (33.6%) and yellow (33.1%) maize from Nigeria as well as maize from China (28.7%) [13,48]. The cellulose content in leaves (39.3%) and stalks (44.9%) of Polish maize was higher than in the corresponding fractions from Italy (35.7% and 36.2%, respectively) [14]. In contrast, cellulose content in husks of tested maize (38.1%) was lower than in Italian maize (40.3%) [14].

### 3.2. The Supermolecular Structure

**Figure 3:**
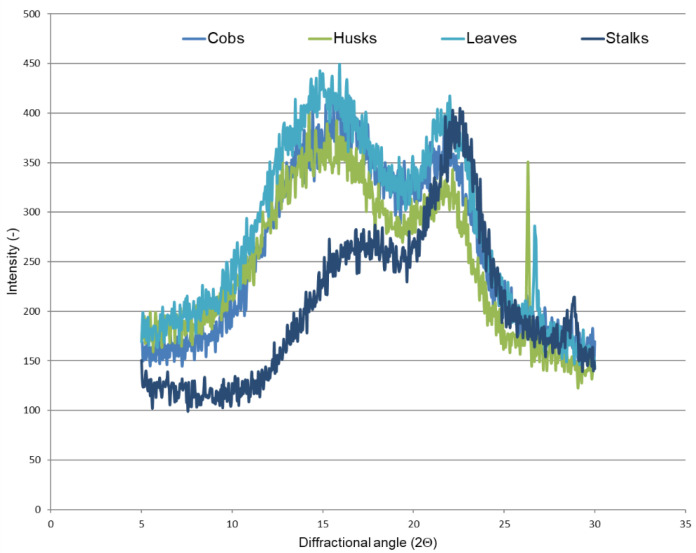
X-ray diffraction patterns of maize stover fractions.

**Figure 4:**
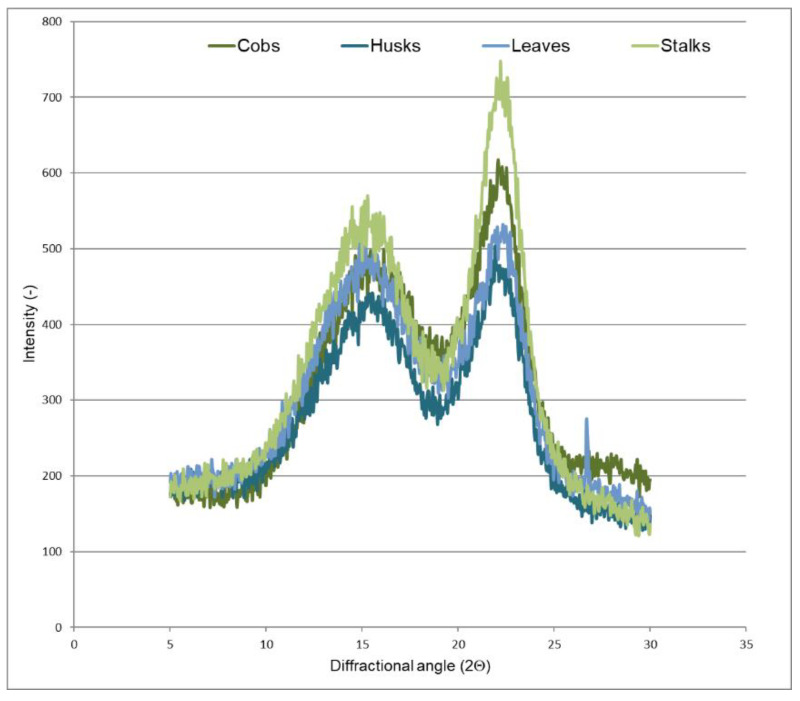
X-ray diffraction patterns of cellulose isolated from maize stover fractions.

Diffractometric analysis was performed to determine the supermolecular structure of lignocellulosic materials. Figure 3 and Figure 4 present X-ray diffractograms of the maize stover fractions and cellulose isolated from these fractions.

It is worth underlining here that the diffraction patterns of the samples show maxima at 2Θ = 15–17° and 22.5° derived from polymorphic cellulose I [49,50]. However, it can be noticed that the curves derived from individual maize fractions are characterized by different intensities of the diffraction maxima, which may indicate changes in the crystallinity of tested samples. Therefore, the next step consisted of calculations of the degree of crystallinity for the maize stover fractions and cellulose isolated from these fractions. The results concerning crystallinity for all the samples are presented in Table 2.

In the case of the individual crude material fractions, the highest degree of crystallinity was found for the stalk fraction (38%), while it was the lowest for the husk fraction (29%). According to available references, the degree of crystallinity for maize stover falls within the range of 25.3–29.8% [51,52]. The high crystallinity index of stalks is associated with their high cellulose content, which was confirmed by our results (Figure 2) and the literature data [33]. Table 2 shows that the highest content of the crystalline phase is found in the fraction from stalks (59%), while it is lowest in husks (48%). It is worth underlining that crystallinity is the effect of ordered areas of cellulose in the crude materials, while other components, e.g., lignin or hemicellulose, are amorphous [53,54].

Our results clearly indicate that the crystallinity of biomass depends on its cellulose and lignin contents. Moreover, literature data suggest that the presence of unstructured components of lignocellulosic materials (e.g., resin and fatty acids) also influence their crystallinity [55,56]. As is well-known, the crystalline fraction of lignocellulosic materials comprise only cellulose since the other main components, i.e., hemicellulose and lignin, as well as resins, fatty acids and low molecular weight components are amorphous. Moreover, these components contribute to the breaking of the hemicellulose–hemicellulose and hemicellulose–cellulose linkages, an increased disorder of cellulose chains, and consequently affect the crystallinity of lignocellulosic materials.

Parameters of crop residues—in Kendall rank correlation coefficient—show a high correlation between the fraction and cellulose content (0.77) and the fraction and lignin content (0.61), while a high negative correlation (−0.78) was observed between crystallinity and nitrogen content (Table 3).

### 3.3. The FTIR Spectra

**Figure 5:**
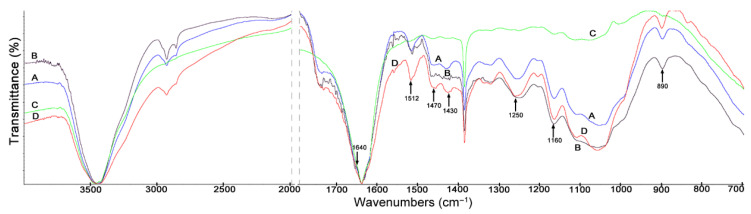
FTIR spectra of maize stover fractions: (**A**) cobs, (**B**) husks, (**C**) leaves, (**D**) stalks.

**Figure 6:**
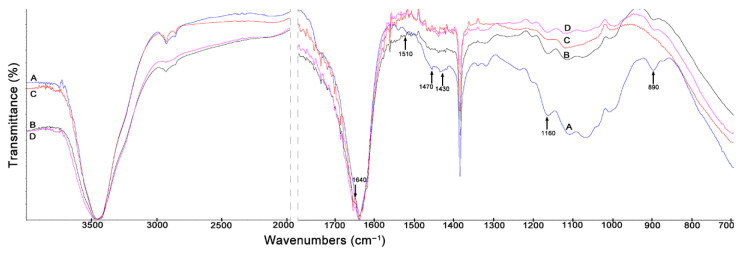
FTIR spectra of cellulose isolated from maize stover fractions: (**A**) cobs, (**B**) husks, (**C**) leaves, (**D**) stalks.

**Figure 7:**
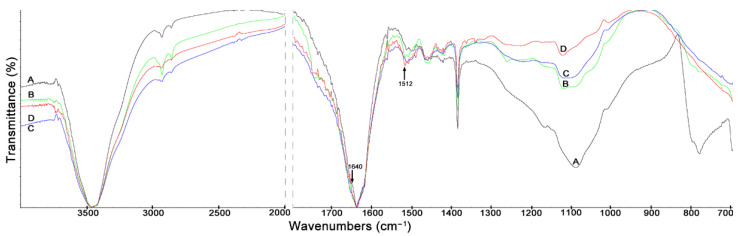
FTIR spectra of lignin isolated from maize stover fractions: (**A**) cobs, (**B**) husks, (**C**) leaves, (**D**) stalks.

Figure 5, Figure 6 and Figure 7 show FTIR spectra of biomass and its main components—cellulose and lignin. The peaks in the FTIR spectra of lignocellulosic biomass samples are assigned in Table 4 to characteristic stretching vibrations of particular groups. For selected bands in the FTIR spectra, the value of relative absorption is given in terms of a lignin-specific band at 1512 cm^−1^ and a cellulose-specific band at 1384 cm^−1^. The highest transmittance was shown at 3400 cm^−1,^ represented by the hydrogen bound stretching bands of O-H groups, possibly originating either from the glucoside linkages of cellulose or the hydroxyphenyl, guaiacyl and syringyl groups of lignin [57].

The FTIR spectrum of stalks is observed with the highest intensity at 1512 cm^−1^ (Figure 5D), which indicates a high content of lignin in this maize fraction (Figure 2). The band in the range of 1500–1700 cm^−1^ represents the aromatic ring stretch [57], while the C=C bond of aromatic skeletal vibrations in lignin appears in the region of 1500–1700 cm^−1^. Symmetric and asymmetric C-H stretching band peak pairs for the representation of crystallinity and amorphous characters, attributed mainly to cellulose molecules (1430 cm^−1^ for crystalline and 890 cm^−1^ for amorphous), were selected in the present study based on earlier data [57]. In the FTIR spectrum of the stalk fraction (Figure 5D), high-intensity bands were observed at 1430 cm^−1^ and 1470 cm^−1^. The peak at 1470 cm^−1^ was due to the C-H and CH_3_- asymmetric scissoring deformations [58]. The highest degree of crystallinity, amounting to 38%, was recorded for this fraction (Table 2), followed by the highest contents of lignin (19.94%) and cellulose (44.87%).

The band intensity at 2895 cm^−1^ indicates a higher hemicellulose concentration, and the band intensity at 1640 cm^−1^ shows a higher lignin concentration in the husk fraction (B) when compared to the other three fractions [57]. It is worth emphasizing that there is no band at 2895 cm^−1^ only in the case of the leaf fraction (Figure 5C). In the FTIR spectrum of leaves (Figure 5C), there is also no band at 1512 cm^−1^, characteristic of the aromatic ring stretch in lignin. This can also be associated with the carbon/nitrogen ratio for the leaf fraction, which is the lowest (45), as shown in Table 1.

On the other hand, in the spectrum of maize stalks (Figure 5D), the band at 1512 cm^−1^ shows the highest intensity. It should be noted that the presence of this band in the stalk fraction (D) or its absence in the leaf fraction (C) is associated with the degree of crystallinity (a high value for the stalk fraction 38%, a low value for the leaf fraction 33%) (Table 2). Moreover, in the leaf fraction spectrum (Figure 5C), there are no bands in the 1430 cm^−1^ and 1470 cm^−1^ range. In the FTIR spectra of cobs, husks and stalks (Figure 5A,B,D), there are bands at 1160 cm^−1^, characteristic for the C-O-C vibration in cellulose and hemicellulose.

In Figure 6, all bands described in Table 4 are observed. The above-described bands appear in the spectra of cellulose isolated from the maize stover fractions with lower or higher intensity. It should be noted that in the FTIR spectrum of cellulose isolated from leaves (Figure 6C), the band at 890 cm^−1^, characteristic of the amorphous cellulose, disappears. The degree of crystallinity determined in cellulose isolated from this fraction, similarly as for the initial fraction, was found to be low (crude material 33%, cellulose 51%).

In the FTIR spectra of lignin isolated from the maize stover fractions (Figure 7), a very broad band of high-intensity is observed at 1040 cm^−1^, especially visible for lignin isolated from cobs (Figure 7A). This band is also present in the FTIR spectra of lignin from the husk, leaf and stalk fractions (Figure 7B–D); however, the intensity of the peak is lower. Naik et al. [58] showed prominent peaks at 1056, 1248, 1506, and 1630 cm^−1^ representing the C-H and O-H bound frequencies being indicators of lignin. The fraction of cobs is characterized by the lowest cellulose content (37.85%) and the relatively low lignin content (13.49%), as shown in Figure 2. Moreover, in the FTIR spectrum of lignin from the cob fraction (Figure 7A), a wide and high peak at 830 cm^−1^ was observed. The peak at 830 cm^−1^ can be assigned to the C-H and CH_3_- asymmetric scissoring deformations [58]. The peak at 1370 cm^−1^ is related to the C-H vibration due to the asymmetric deformation of cellulose and lignin [52].

## 4. Conclusions

The results presented in this paper indicate that the fractions of maize stover harvested in Poland showed differences in the chemical composition and supermolecular structure. Kendall’s statistical analysis indicated a strong correlation between the maize fractions and cellulose content as well as lignin content and individual fractions. The stalks were characterized by the highest cellulose and lignin contents. This fraction also showed the highest degree of crystallinity and the most significant changes in FTIR spectra.

The basic parameter used when assessing the suitability of substrates for the methane fermentation process is their digestibility. This parameter depends on the chemical characteristics of biomass. Knowledge of the chemical characteristics and supermolecular structure of the substrate and appropriate physicochemical preparation of the material to the fermentation process will facilitate and promote the availability of components for microorganisms, which will likely provide a higher yield of biogas, which consequently means higher energy production.

Presented results may also be valuable not only for the design of the biogas fermentation process but also for the indication of potential utilization of individual maize fractions in the wood industry during the production of nanocellulose and nanometric fillers based on lignocellulose biomass or as additives in feeds for ruminants.

## Figures and Tables

**Table 1:** Chemical composition of maize stover fractions.

Fraction	C * (%)	N * (%)	C:N *	H (%)	O (%)
Cobs	44.81 ^a^ ± 0.04	0.53 ^b^ ± 0.04	84 ^b^ ± 7	5.93 ^a^ ± 0.04	45.03 ^a^ ± 1.00
Husks	43.79 ^b^ ± 0.17	0.53 ^b^ ± 0.06	83 ^b^ ± 8	6.00 ^a^ ± 0.26	43.15 ^ab^ ± 0.92
Leaves	43.31 ^c^ ± 0.06	0.96 ^a^ ± 0.05	45 ^c^ ± 2	5.72 ^a^ ± 0.04	41.91 ^ab^ ± 0.49
Stalks	43.73 ^bc^ ± 0.11	0.30 ^c^ ± 0.00	147 ^a^ ± 1	5.55 ^a^ ± 0.07	41.35 ^b^ ± 0.78
n	3	3	3	3	3

The average values (n) ± standard deviation; average values in columns labeled with the same superscript (a, b, c) are not significantly different according to the HSD Tukey’s test (ANOVA) for the investigated factors. * data according to [3].

**Table 2:** The degree of crystallinity of maize stover fractions and cellulose isolated from these fractions.

Fraction	Crude Material (%)	Cellulose (%)
Cobs	32	54
Husks	29	48
Leaves	33	51
Stalks	38	59

**Table 3:** Kendall’s τ coefficient between maize stover fractions, chemical composition and degree of crystallinity.

	Fraction	Cellulose	Lignin	C	N	C:N	H	O	Degree of Crystallinity
**Fraction**	-	-	-	-	-	-	-	-	-
**Cellulose**	0.77	-	-	-	-	-	-	-	-
**Lignin**	0.61	0.71	-	-	-	-	-	-	-
**C**	−0.54	−0.36	−0.21	-	-	-	-	-	-
**N**	−0.15	0.00	−0.00	−0.21	-	-	-	-	-
**C:N**	0.07	0.07	0.07	0.28	−0.93	-	-	-	-
**H**	−0.77	−0.57	−0.71	0.50	0.28	−0.21	-		-
**O**	−0.84	−0.71	−0.57	0.50	0.00	0.07	0.57	-	-
**Degree of crystallinity**	0.31	0.21	−0.07	0.00	−0.78	0.71	−0.21	−0.21	-
**Degree of crystallinity of isolated cellulose**	0.31	0.18	−0.04	0.04	−0.76	0.69	−0.25	−0.18	0.98

**Table 4:** Assignment of selected FTIR bands of functional groups in lignocellulosic stover biomass samples and relative absorbance value for each fraction of maize stover.

Assignment of Selected FTIR Bands of Functional Groups		Relative Absorbance Value for Each Fraction of Maize Stover							
Wavenumber (cm^−1^)	Group and Their Stretching Vibrations	Cobs		Husks		Leaves		Stalks	
		Maize Stover *	Cellulose **	Maize Stover *	Cellulose **	Maize Stover *	Cellulose **	Maize Stover *	Cellulose **
2930	CH_2_ and CH_3_ asymmetric and symmetric stretching vibrations	1.250	0.575	1.364	0.684	1.000	0.742	1.057	2.172
1640	C=C of aromatic vibrations in lignin	3.125	1.200	3.364	1.684	1.800	1.871	2.019	3.724
1470	C-H deformation stretching in lignin and xylan	1.250	0.528	1.409	0.649	1.000	0.390	1.151	1.552
1430	C-H crystalline cellulose	1.344	0.593	1.473	0.667	1.200	0.410	1.226	1.448
890	C-H stretching out of the plane of the aromatic ring, and asymmetric, out of phase ring stretching in cellulose, CH amorphous cellulose	1.000	1.875	3.873	0.561	0.000	0.000	0.981	0.655

* Absorbance compared to the value at 1512 cm^−1^, ** absorbance compared to the value at 1384 cm^−1^.

## Data Availability

The data reported in this study can be available by requesting the authors.
